# Association between triglyceride-glucose index and obstructive sleep apnea severity in hypertensive patients with co-existing OSA: a cross-sectional study

**DOI:** 10.3389/fendo.2025.1669661

**Published:** 2025-09-29

**Authors:** Yan Li, Lu Zhang, Lu Zhai, Limantian Wang, Shudan Deng, Xiaolin Hao, Ying Zhang, Xiaoling Gao

**Affiliations:** ^1^ The Second Clinical Medical College of Shanxi Medical University, Taiyuan, Shanxi, China; ^2^ Department of Respiratory and Critical Care Medicine, The Second Hospital of Shanxi Medical University, Taiyuan, Shanxi, China

**Keywords:** triglyceride-glucose index, obstructive sleep apnea, hypertension, insulin resistance, biomarker

## Abstract

**Study objectives:**

To evaluate the association between the triglyceride-glucose (TyG) index and obstructive sleep apnea (OSA) severity in hypertensive patients with comorbid OSA, particularly in non-obese subgroups.

**Methods:**

This cross-sectional study consecutively enrolled 653 hypertensive patients with snoring and excessive daytime sleepiness from the Second Hospital of Shanxi Medical University between 2022 and 2023. After confirming OSA diagnosis by polysomnography, 562 eligible participants were stratified into mild/moderate/severe OSA groups. The TyG index was calculated as ln[fasting triglycerides (mg/dL) × glucose (mg/dL)/2]. Multivariable ordinal logistic regression was performed to identify predictors of OSA severity, with subgroup analyses stratified by BMI. Linear regression was employed to examine the association between the TyG index and the apnea-hypopnea index (AHI).

**Results:**

In the fully adjusted model, the TyG index showed the strongest independent association with OSA severity progression (OR = 1.885, 95%CI:1.107-3.209), demonstrating greater explanatory value than BMI based on standardized β coefficients. This association demonstrated striking phenotypic specificity, with significant correlation restricted to non-obese individuals (adjusted OR = 2.804, 95%CI:1.547-5.083) versus obese counterparts. Stratification by TyG tertiles revealed progressive AHI escalation with increasing tertiles (β = 8.265 per tertile, *P* < 0.001), indicating a dose-response relationship.

**Conclusions:**

The TyG index surpasses conventional obesity metrics in stratifying OSA severity among hypertensive patients with OSA. These findings support its utility as a pathophysiology-guided risk stratification tool for OSA-related cardiometabolic complications in hypertension management.

## Background

Obstructive sleep apnea (OSA), the predominant sleep-associated respiratory disorder worldwide, poses substantial healthcare burdens. Global epidemiological surveillance estimates that 936 million individuals are affected by OSA, including 425 million with moderate-to-severe disease requiring therapeutic intervention (1). Notably, OSA exhibits a pronounced comorbidity profile with hypertension, atrial fibrillation, and heart failure (2–4). A critical bidirectional pathophysiological relationship exists between OSA and hypertension: epidemiological studies indicate that nearly 50% of OSA patients have concurrent hypertension, while 30%–50% of hypertensive individuals have comorbid OSA (5, 6). This interdependent comorbidity not only complicates clinical management but also substantially elevates the risk of adverse cardiovascular events (7, 8).

Although the AHI remains the gold standard for quantifying OSA severity, evidence reveals its limited correlation with metabolic pathophysiology, particularly in reflecting insulin resistance progression and lipid profile abnormalities (9, 10).These metabolic alterations are strongly associated with hypertension-mediated target organ damage. Notably, IR and related metabolic abnormalities have been demonstrated to independently correlate with both end-organ injury and adverse cardiovascular outcomes in OSA populations (11). In patients with coexisting hypertension and OSA, metabolic dysregulation may accelerate disease progression through mechanisms involving inflammatory activation and oxidative stress (12). Consequently, identifying biomarkers capable of simultaneously capturing respiratory dysfunction and metabolic perturbations holds critical clinical significance for risk stratification and therapeutic targeting in this high-risk cohort.

The TyG index, calculated from fasting serum lipid and glycemic parameters, has emerged as a reliable surrogate marker for quantifying insulin resistance in modern metabolic profiling studies (13). Clinical studies have demonstrated elevated TyG index levels in both hypertensive and OSA populations (14, 15). A cross-sectional analysis identified the TyG index as the strongest independent indicator of incident hypertension (16), while a prospective cohort study established its association with an increased risk of OSA development (17). Nevertheless, the clinical utility of the TyG index in stratifying OSA progression among hypertensive patients with concurrent OSA has not been systematically investigated, underscoring an unaddressed mechanistic link between impaired metabolic homeostasis and cardiovascular-pulmonary multimorbidity.

This study proposes the TyG index as a biomarker associated with OSA severity in patients with hypertension and OSA, addressing current evidence limitations in this population. Employing a cross-sectional design, we systematically evaluate the association between TyG index levels and polysomnographic parameters. These findings may not only advance risk stratification protocols but also inform the development of individualized therapeutic strategies for this high-risk population, thereby bridging the clinical translation gap in cardiometabolic-sleep disorder comorbidity management.

## Methods

### Subjects and study design

**Figure 1:**
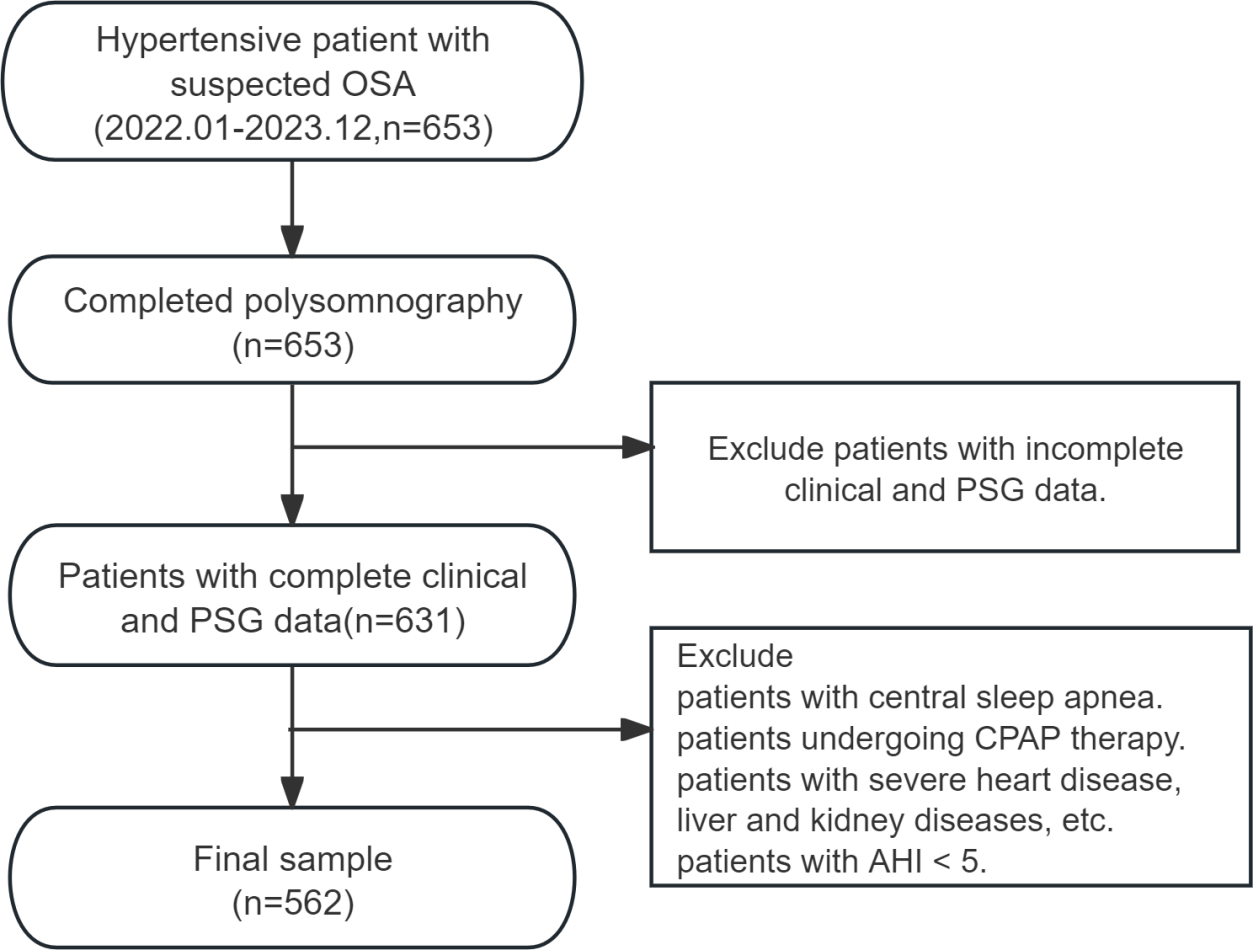
Flowchart of study sample selection.

A cross-sectional study was conducted involving patients with hypertension and symptoms such as snoring and daytime sleepiness, who were admitted to the Second Hospital of Shanxi Medical University between January 2022 and December 2023. The study cohort comprised six hundred and fifty-three consecutively enrolled participants meeting predefined inclusion criteria. Polysomnographic monitoring was subsequently performed, and based on the results, hypertensive patients with OSA were categorized into three groups: HTN with mild OSA, HTN with moderate OSA, and HTN with severe OSA (**Figure 1**).

Hypertension diagnosis required fulfillment of either criterion: (1) sustained elevation of blood pressure (SBP ≥140 mmHg and/or DBP ≥90 mmHg) across three consecutive measurements, or (2) physician-confirmed hypertension diagnosis with active antihypertensive regimen. OSA diagnosis required: 1) Clinical manifestations including nocturnal snoring, excessive daytime sleepiness, sleep-related choking, or cardiometabolic comorbidities (e.g., coronary artery disease, type 2 diabetes); 2) Polysomnography demonstrating obstructive apnea-hypopnea index (AHI) ≥5 events/hour. OSA confirmation mandated fulfillment of both clinical and PSG criteria according to American Academy of Sleep Medicine guidelines.

The exclusion criteria were: 1) Sleep disorders resulting from psychiatric conditions; 2) A definite diagnosis of secondary hypertension unrelated to OSA; 3) Acute or chronic heart failure, severe arrhythmias, valvular heart disease, cardiomyopathy, aortic dissection, and other related conditions; 4) Acute or chronic infections, anemia, hemorrhagic disorders, autoimmune diseases, severe liver or kidney dysfunction, or malignant tumors; 5) Patients currently receiving continuous positive airway pressure (CPAP) therapy.

The Institutional Review Board at Shanxi Medical University’s Second Affiliated Hospital granted ethical clearance for this investigation(2025YX181), with documented informed consent obtained from all study participants.

### Polysomnographic monitoring

Overnight polysomnography (PSG) was performed using the Embla N7000 system (Natus Medical Incorporated, Pleasanton, CA) following standardized protocols. Patients were instructed to abstain from alcohol, caffeine-containing beverages, and psychotropic medications for 24 hours preceding the examination. EEG recordings were obtained through surface electrodes positioned in accordance with the International 10–20 system configuration. Respiratory monitoring included dual-channel pressure transducer and thermistor sensors for simultaneous nasal/oral airflow detection. Peripheral capillary oxygen saturation (SpO2) was continuously monitored using a Nonin 8000R fingertip oximeter (Nonin Medical, Plymouth, MN).All PSG data underwent blinded analysis by two board-certified sleep specialists using the American Academy of Sleep Medicine scoring criteria (version 2.3; 2016). Inter-rater reliability was maintained through consensus-based resolution of scoring discrepancies, with unresolved cases being adjudicated by a senior sleep specialist (>15 years clinical experience).

### Data collection and measurements

Demographic parameters (gender, age, smoking history, alcohol intake) and clinical identifiers were prospectively documented. Venous blood specimens were obtained following ≥8 hours of nocturnal fasting for multianalyte assessment, quantifying: hematologic indices (WBC, Hb, PLT), hepatic function panel (ALT, AST, TBIL, DBIL), renal markers (UREA, Scr), lipid metabolism profile (TC, TG, HDL, LDL), glycemic regulators (FBG, HbA1c), and coagulation parameter D-Di. Insulin resistance evaluation employed the validated TyG algorithm: ln[(TG × FBG)/2], with lipid and glucose measurements standardized in mg/dL.

Standard polysomnography captured 18 core variables: Total Sleep Time (TST), Sleep Latency (SL), Sleep Efficiency (SE), REM Latency (RL), Oxygen Saturation [average (AvgSpO_2_) and minimum (LSpO_2_)], Heart Rate (AvgHR), Sleep Stage Distribution [REM% and NREM phases (N1%, N2%, N3%)], Apnea Duration (LAD), and Respiratory Event Indices [HI, TAI, OAI, CAI, MAI, AHI].

### Statistical analysis

Statistical analyses and graphical visualizations in this study were performed utilizing R software (version 4.2.1) and IBM SPSS Statistics (version 25.0). Continuous variables underwent distribution normality evaluation using Shapiro-Wilk methodology. Parametric data adopted mean ± SD summarization, whereas non-parametric measures utilized median (IQR) descriptors. Discrete variables employed frequency enumeration with proportional quantification. Group comparisons among HTN patients with mild/moderate/severe OSA utilized one-way ANOVA with Bonferroni *post hoc* correction (normal data), Kruskal-Wallis test(non-normal data), and Chi- square/Fisher’s exact tests (categorical variables). OSA severity associated factors were identified through univariate screening followed by multivariate ordinal logistic regression, with effects quantified by adjusted ORs (95% CI). TyG tertile-stratified analyses included ANOVA/Kruskal-Wallis tests for baseline comparisons, Spearman correlations for sleep parameter associations, and linear regression models for AHI determinants, with two-tailed *P* < 0.05 defining significance.

## Results

### Clinical and biochemical characteristics in HTN with mild OSA, moderate OSA, and severe OSA groups

This cross-sectional study enrolled 562 patients with concomitant hypertension and OSA, comprising 415 males (73.8%) and 147 females (26.2%). Participants were stratified into three groups based on OSA severity: 99 patients with hypertension and mild OSA (HTN-mild OSA), 111 with hypertension and moderate OSA (HTN-moderate OSA), and 352 with hypertension and severe OSA (HTN-severe OSA). The mean ages across groups were 53.34 ± 15.59 years, 55.41 ± 13.90 years, and 52.30 ± 13.31 years (**Table 1**).

**Table 1:** Demographic and clinical characteristics of participantsby the severity of OSA.

Variable	HTN-mild OSA(n=99)	HTN-moderate OSA(n=111)	HTN-severe OSA(n=352)	P value
Male,n(%)	68(68.7%)	83(74.8%)	264(75.0%)	0.437
Age(years)	53.34 ± 15.59	55.41 ± 13.90	52.30 ± 13.31	0.116
BMI(kg/m^2^)	26.03(24.24-29.35)	26.85(25.09-30.12)	29.04(26.46-31.94)	<0.001
Smoking,n(%)	48(48.5%)	59(53.2%)	170(48.3%)	0.661
Drinking,n(%)	60(60.6%)	74(66.7%)	207(58.8%)	0.335
WBC(10^9/L)	7.03(5.61-8.24)	6.63(5.78-8.24)	6.91(5.55-8.25)	0.702
Hb(g/L)	149.55 ± 20.63	149.04 ± 17.72	149.19 ± 23.18	0.985
PLT(10^9/L)	237(199-289)	227(180-267)	224(182-270)	0.078
ALT(U/L)	28.70(15.20-38.80)	23.70(14.90-35.60)	25.65(18.33-40.78)	0.116
AST(U/L)	22.50(17.80-27.50)	21.60(17.60-27.70)	22.65(18.80-30.10)	0.183
TBIL(umol/L)	13.40(10.00-18.40)	13.00(9.70-18.20)	14.25(10.63-17.98)	0.541
DBIL(umol/L)	2.70(2.00-3.10)	2.60(1.85-3.40)	2.60(1.90-3.50)	0.865
UREA(mmol/L)	5.10(4.36-6.20)	5.10(4.10-6.00)	5.45(4.50-6.30)	0.054
Scr(umol/L)	70.00(60.00-83.76)	72.00(61.19-81.00)	74.00(61.00-84.78)	0.192
TC(mmol/L)	4.58(3.60-5.25)	4.52(3.58-5.05)	4.58(3.91-5.26)	0.075
TG(mmol/L)	1.73(1.19-2.30)	1.76(1.23-2.44)	2.14(1.48-2.69)	0.001
HDL(mmol/L)	1.10(0.90-1.29)	1.12(0.89-1.24)	1.12(0.91-1.28)	0.967
LDL(mmol/L)	2.06(1.60-2.65)	2.14(1.62-2.64)	2.24(1.68-2.86)	0.077
FBG(mmol/L)	5.26(4.68-5.87)	5.16(4.77-5.93)	5.67(5.03-6.67)	<0.001
TyG index	8.92(8.52-9.28)	9.04(8.55-9.28)	9.17(8.79-9.50)	<0.001
D-Di(ng/mL)	62.00(0.98-109.00)	61.00(28.00-114.00)	64.00(3.94-116.00)	0.451
HbA1c(%)	6.00(5.60-6.40)	5.90(5.60-6.40)	6.20(5.70-6.78)	0.004

**Figure 2:**
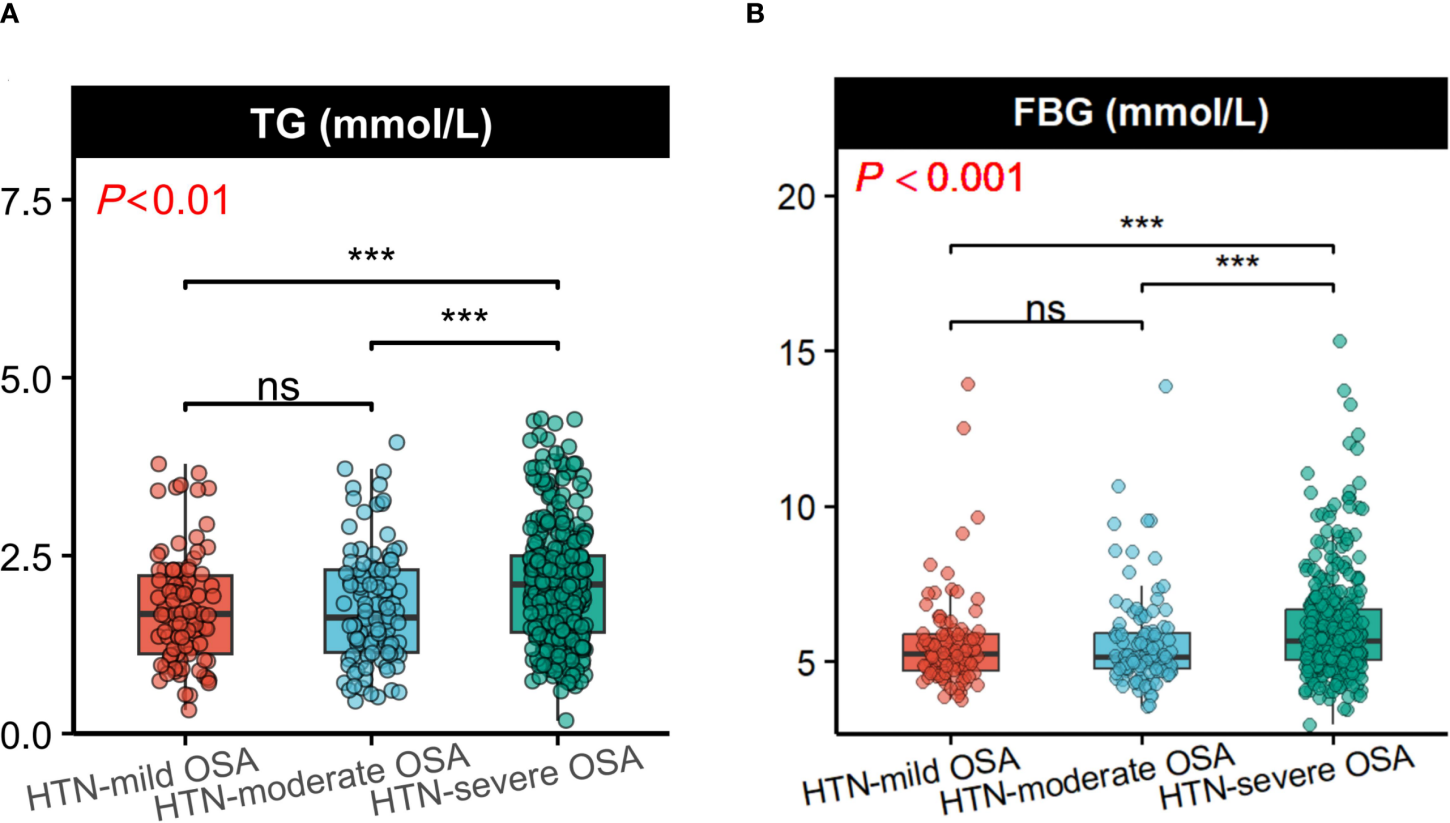
Comparison of TG and FBG levels among the three groups.

Comparative analysis revealed no statistically significant intergroup differences in smoking history, alcohol consumption, or the following clinical parameters: WBC, Hb, PLT, ALT, AST, TBIL, DBIL, UREA, Scr, TC, HDL, LDL, and D-dimer levels (all *P*>0.05).Notably, the HTN-severe OSA group demonstrated significantly elevated metabolic indices compared to other groups, including BMI, TG, FBG, TyG index (*P* < 0.001), and HbA1c (**Table 1**, **Figure 2**). These findings suggest a distinct metabolic profile associated with OSA severity in hypertensive patients.

### Ordinal logistic regression analysis of OSA severity in HTN-OSA comorbidity

**Figure 3:**
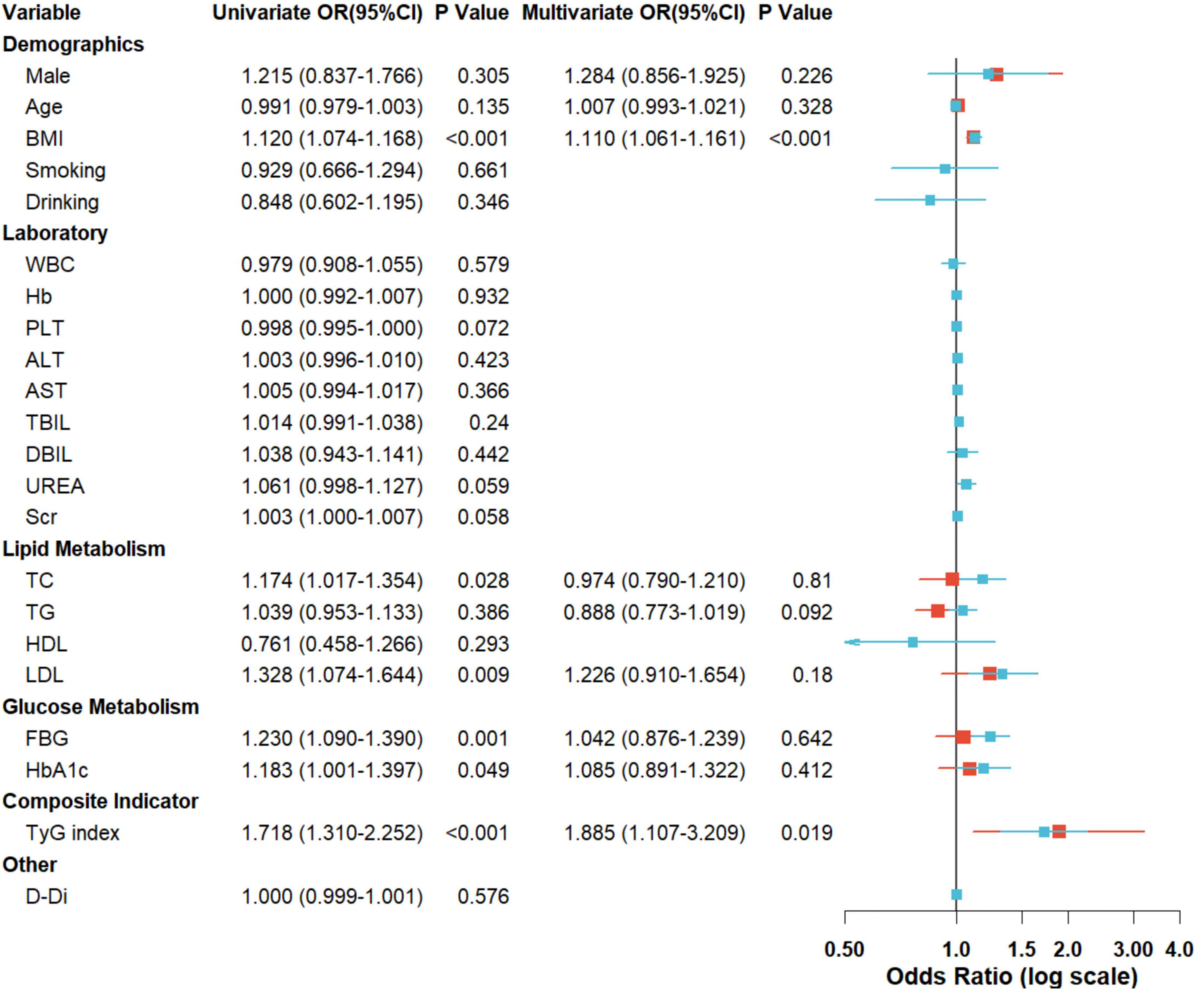
Forest plot of risk factors for OSA severity in HTN-OSA comorbidity.

We employed ordinal logistic regression to identify determinants of OSA severity progression in patients with comorbid hypertension and OSA. Initial univariate analysis revealed significant associations between OSA severity and BMI, TC, LDL-C, FBG, TyG index, and HbA1c(all *P* < 0.05) (**Figure 3**). However, multivariate regression demonstrated that only TyG index and BMI retained independent associations with OSA severity stratification. Specifically, each 1-unit elevation in TyG index corresponded to an 88.5% increased likelihood of advancing to higher OSA severity grades (OR = 1.885, 95%CI 1.107-3.209, *P* = 0.019), while a 1 kg/m² increment in BMI was associated with an 11.0% risk escalation (OR = 1.110, 95%CI 1.061-1.161, *P* < 0.001) (**Figure 3**). Notably, TyG index exhibited a substantially greater effect magnitude than conventional metabolic markers, suggesting that insulin resistance may play a central role in the pathophysiological progression of OSA. Importantly, traditional metabolic parameters including TC,TG, LDL-C, FBG, and HbA1c lost statistical significance in multivariable modeling (*P*> 0.10), suggesting that the TyG index holistically encapsulates the pathophysiological interplay between glucose-lipid dysregulation and OSA progression.

### ROC curve analysis of TyG index

**Figure 4:**
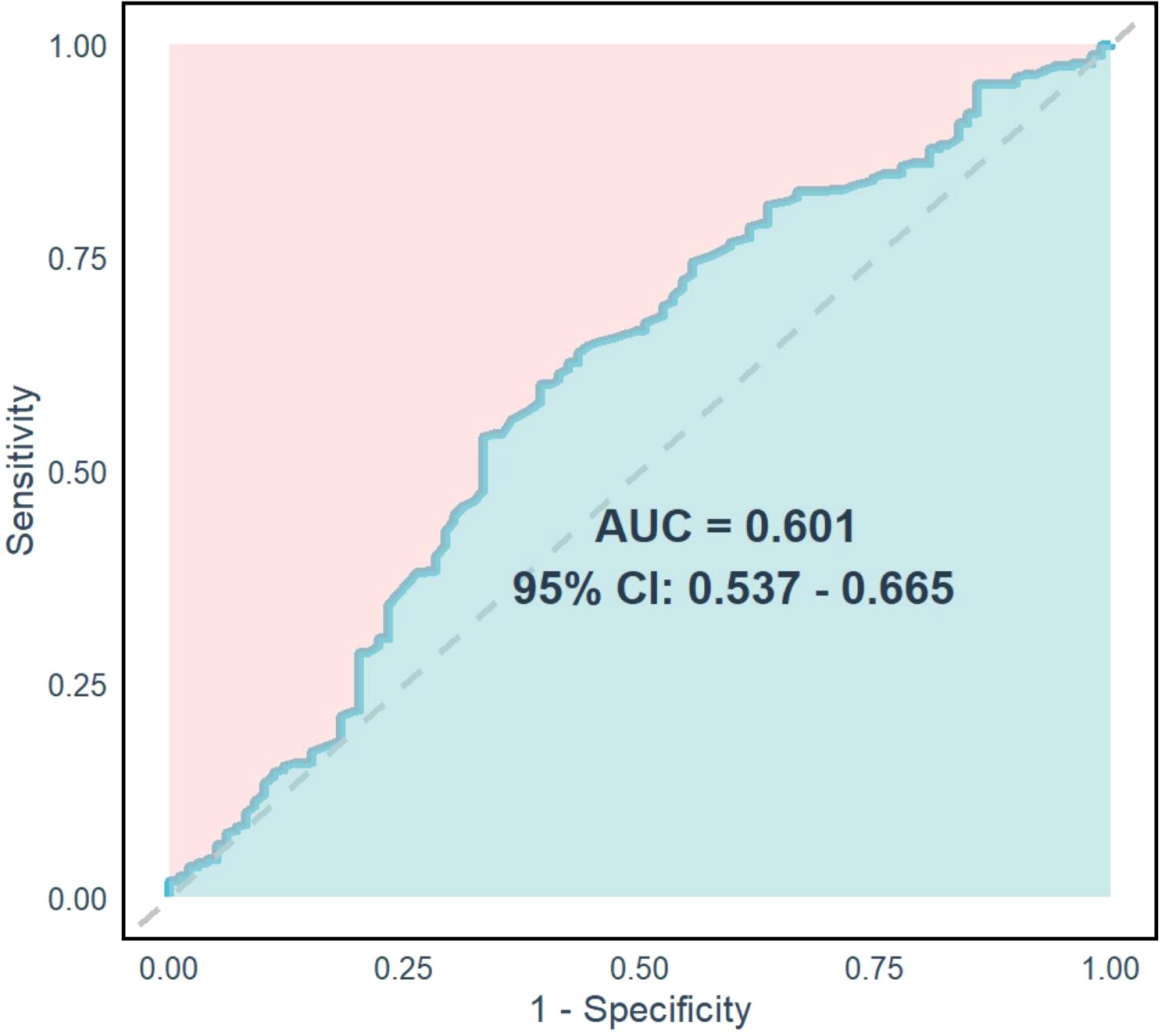
ROC curve analysis of TyG index.

ROC curve analysis was performed to evaluate the diagnostic performance of the TyG index in distinguishing between middle-severe OSA (event group, 1) and mild OSA (non-event group, 0). The AUC was 0.601 (95% CI 0.537-0.665, *P* = 0.002), indicating moderate discrimination. The sensitivity was 0.644, suggesting the TyG index correctly identified 64.4% of middle-severe OSA cases. However, the specificity was low at 0.356, reflecting a higher rate of false positives. The Youden’s index was 8.9569, indicating a suboptimal balance between sensitivity and specificity (**Figure 4**). These results highlight that while the TyG index has statistical significance, its diagnostic ability for OSA severity remains limited, warranting further optimization.

### Subgroup analysis based on BMI categories

**Figure 5:**
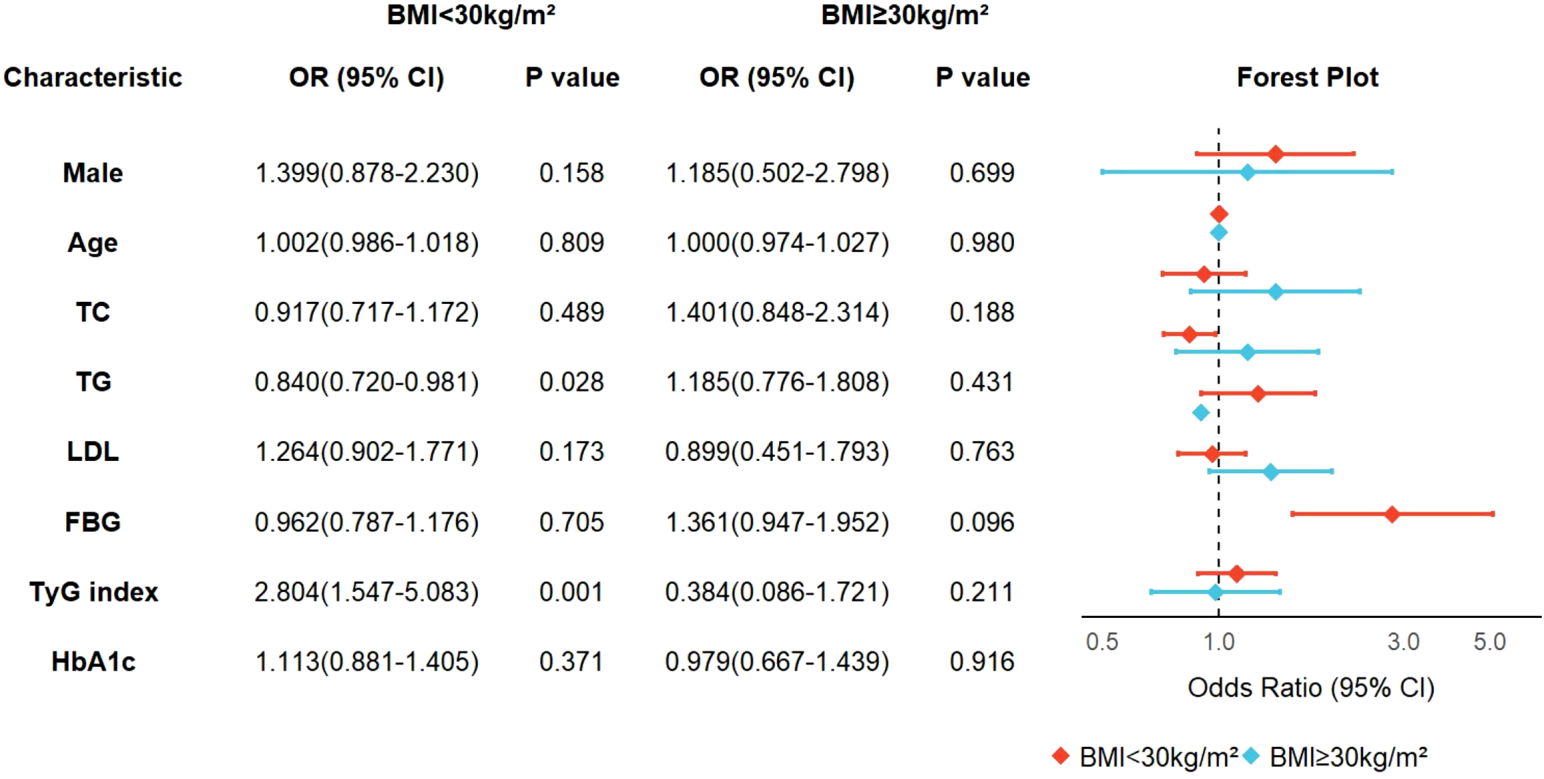
Subgroup analysis based on BMI categories.

Subsequent subgroup analyses stratified by BMI revealed non-significant interaction terms (*P*>0.05) in the main effect model, suggesting no statistically significant joint effect between TyG index and BMI on the outcome variable. This observation implies independent rather than interactive effects of TyG index and BMI on OSA severity progression. Notably, in non-obese individuals (BMI<30 kg/m²), TyG index demonstrated a significant positive association with OSA severity (adjusted OR = 2.804, 95% CI:1.547-5.083; *P* = 0.001), whereas this relationship was abolished in the obese subgroup (BMI ≥ 30 kg/m²; OR = 0.384, 95% CI:0.086-1.721; *P* = 0.211). Intriguingly, triglyceride levels exhibited a paradoxical protective association with OSA severity exclusively in non-obese participants (OR = 0.840, 95% CI:0.720-0.981; *P* = 0.028), potentially indicating the existence of population-specific lipid redistribution mechanisms that modulate cardiometabolic risk profiles in this subpopulation (**Figure 5**).

### Clinical and biochemical characteristics by TyG index

To elucidate the role of the TyG index in patients with coexisting hypertension and OSA, we stratified participants into tertiles based on TyG index values: Tertile 1 (n=188, TyG index ≤ 8.84), Tertile 2 (n=192, 8.85 < TyG index ≤ 9.28), and Tertile 3 (n=182, TyG index > 9.29). Median TyG indices with interquartile ranges were 8.51 (8.24 - 8.69), 9.12 (9.00 - 9.21), and 9.66 (9.45 - 9.88) across the respective tertiles. Significant intergroup differences were observed in age, BMI, alcohol consumption, WBC, Hb, PLT, ALT, AST, DBIL, TG, TC, LDL, HDL, FBG, TyG index, D-Di, and HbA1c (all *P* < 0.05) (**Table 2**).

**Table 2:** Demographic and clinical characteristics of participants by TyG index.

Variable	Tertile 1(n=188)	Tertile 2(n=192)	Tertile 3(n=182)	P value
Male,n(%)	142(75.5%)	131(68.2%)	142(73.8%)	0.080
Age(years)	54.73 ± 14.32	53.76 ± 14.79	50.71 ± 12.08	0.009
BMI(kg/m2)	26.72(24.45-30.49)	28.40(25.96-31.51)	29.00(26.57-31.63)	<0.001
Smoking,n(%)	91(48.4%)	105(54.7%)	81(44.5%)	0.138
Drinking,n(%)	119(63.3%)	133(69.3%)	89(48.9%)	<0.001
WBC(10^9/L)	6.79(5.41-8.13)	6.87(5.55-8.00)	7.04(5.97-8.63)	0.041
Hb(g/L)	148(135-161)	149(137-165)	154(139-165)	0.031
PLT(10^9/L)	218.18 ± 66.48	229.16 ± 60.27	243.55 ± 65.25	0.001
ALT(U/L)	22.35(14.70-34.30)	25.25(17.05-38.18)	30.54(21.68-46.28)	<0.001
AST(U/L)	21.40(17.90-26.05)	22.80(18.63-28.78)	23.75(18.75-32.35)	0.012
TBIL(umol/L)	14.20(10.50-18.48)	13.55(10.00-16.80)	14.05(10.45-18.80)	0.353
DBIL(umol/L)	2.88(2.13-3.60)	2.65(1.83-3.20)	2.50(1.78-3.33)	0.001
UREA(mmol/L)	5.50(4.30-6.38)	5.40(4.50-6.19)	5.20(4.30-6.22)	0.595
Scr(umol/L)	73.00(61.00-85.00)	74.00(61.03-83.76)	72.98(61.00-83.76)	0.782
TC(mmol/L)	3.96(3.21-4.92)	4.58(4.19-5.13)	4.86(4.39-5.62)	<0.001
TG(mmol/L)	1.18(0.94-1.45)	2.09(1.82-2.30)	3.04(2.46-3.82)	<0.001
HDL(mmol/L)	1.13(0.95-1.28)	1.12(0.94-1.27)	1.00(0.84-1.28)	0.002
LDL(mmol/L)	1.96(1.45-2.54)	2.24(1.81-2.81)	2.24(1.77-2.83)	<0.001
FBG(mmol/L)	5.02(4.51-5.52)	5.58(4.97-6.01)	6.25(5.30-7.70)	<0.001
TyG index	8.51(8.24-8.69)	9.12(9.00-9.21)	9.66(9.45-9.88)	<0.001
D-Di(ng/mL)	76.00(21.79-131.00)	64.50(8.25-116.25)	51.00(0.92-100.75)	0.006
HbA1c(%)	5.90(5.50-6.31)	6.10(5.70-6.50)	6.30(5.78-6.95)	<0.001

### Sleep monitoring parameters of participants grouped by TyG index

To further elucidate the relationship between the TyG index and hypertensive patients with co-existing OSA, we conducted a comparative analysis of polysomnographic parameters across TyG index tertiles. The TyG-stratified cohorts exhibited stepwise elevation in respiratory disturbance severity, with median AHI progressively increasing across tertiles [Tertile 1: 30.58(15.14-51.65) vs Tertile 2: 37.46(22.55-66.57) vs Tertile 3: 46.45(29.02-71.79); *P* < 0.001]. Concurrent graded impairment manifested in nocturnal hypoxemia profiles, evidenced by marked attenuation of LSpO_2_in the highest TyG tertile[Tertile 3: 77.00(65.50-83.00)vs Tertile 2: 77.00(66.25-84.00) vs Tertile 1:80.50(73.25-86.00), *P* < 0.001]. Furthermore, significant statistical differences were observed among the three groups in parameters such as average heart rate, longest apnea duration, total apnea Index, and obstructive Apnea Index (**Table 3**).

**Table 3:** Sleep monitoring parameters of participants grouped by TyG index.

Variable	Tertile 1(n=188)	Tertile 2(n=192)	Tertile 3(n=182)	P value
Total Sleep Time(h)	6.35 ± 1.26	6.45 ± 1.26	6.50 ± 1.27	0.529
Sleep Efficiency(%)	77.50(64.08-86.00)	79.00(67.00-87.88)	78.65(71.95-87.35)	0.107
Sleep Latency(min)	7.00(3.00-14.5)	7.10(3.10-18.58)	7.05(3.00-15.03)	0.510
REM Latency(min)	96.25(62.25-143.25)	97.00(68.13-158.63)	100.00(70.5-146.75)	0.536
AvgSpO_2_(%)	93.00(91.40-94.38)	92.35(90.00-94.00)	92.25(90.00-94.00)	0.057
LSpO_2_(%)	80.50(73.25-86.00)	77.00(66.25-84.00)	77.00(65.50-83.00)	<0.001
Average Heart Rate	66.0(60.25-73.0)	68.0(62.0-74.0)	68.0(64.0-74.0)	0.040
REM(%)	17.44 ± 7.68	17.31 ± 7.26	17.90 ± 6.17	0.668
N1(%)	27.85(18.90-41.18)	24.90(18.93-40.23)	29.10(19.98-44.08)	0.332
N2(%)	41.80(34.50-50.16)	42.70(32.78-50.05)	40.15(31.00-50.35)	0.420
N3(%)	9.85(3.63-15.20)	8.25(2.00-14.50)	8.45(2.65-13.83)	0.449
Longest Apnea Duration(s)	38.00(24.00-59.25)	43.80(25.00-68.75)	47.50(26.00-72.00)	0.037
Hypopnea Index	15.41(10.11-24.65)	19.35(9.15-28.05)	17.63(10.75-28.93)	0.169
Total Apnea Index	8.70(1.93-27.83)	13.15(2.93-43.54)	23.00(4.30-55.81)	<0.001
Obstructive Apnea Index	6.00(1.15-23.35)	12.00(2.45-34.55)	16.60(3.08-39.33)	<0.001
Mixed Apnea Index	0.00(0.00-1.20)	0.00(0.00-1.70)	0.20(0.00-3.00)	0.007
Central Apnea Index	0.05(0.00-1.10)	0.00(0.00-1.00)	0.30(0.00-1.50)	0.083
AHI	30.58(15.14-51.65)	37.46(22.55-66.57)	46.45(29.02-71.79)	<0.001

### Correlation between TyG index and AHI

**Figure 6:**
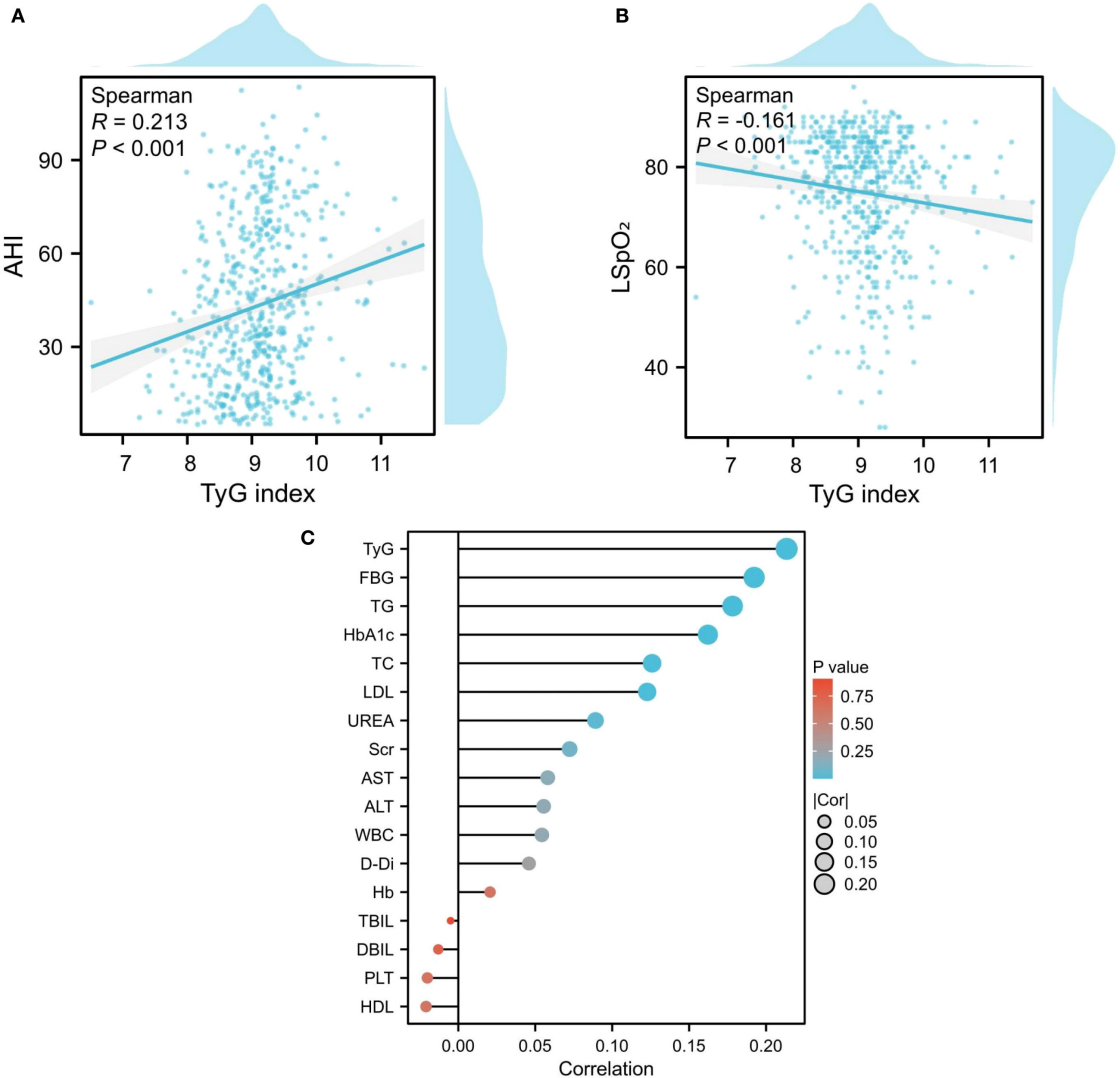
Correlation between sleep monitoring indicators and biochemical parameters. **(A, B)** Association Between TyG Index and AHI/LSPO2; **(C)** The correlation between AHI and laboratory indicators.

The TyG index demonstrated significant correlations with key polysomnographic markers of OSA severity. Notably, it exhibited a positive association with the AHI(ρ=0.214, *P* < 0.001), alongside progressive correlations with respiratory event metrics including sleep efficiency (ρ=0.096, *P* = 0.023), longest apnea duration (ρ=0.095, *P* = 0.024), total apnea index (ρ=0.168, *P* < 0.001), obstructive apnea index (ρ=0.166, *P* < 0.001), and mixed apnea index (ρ=0.098, *P* = 0.020) (**Figure 5**). Concurrently, inverse correlations emerged with oxygenation parameters, showing reduced AvgSpO_2_(ρ=-0.087, *P* = 0.040) and LSpO_2_(ρ=-0.161, *P* < 0.001).Furthermore, the study found a positive correlation between AHI and laboratory indicators such as UREA, TC, TG, LDL, FBG, TyG, and HbA1c (**Figure 6**).

### Univariate and multivariate linear regression of AHI

**Figure 7:**
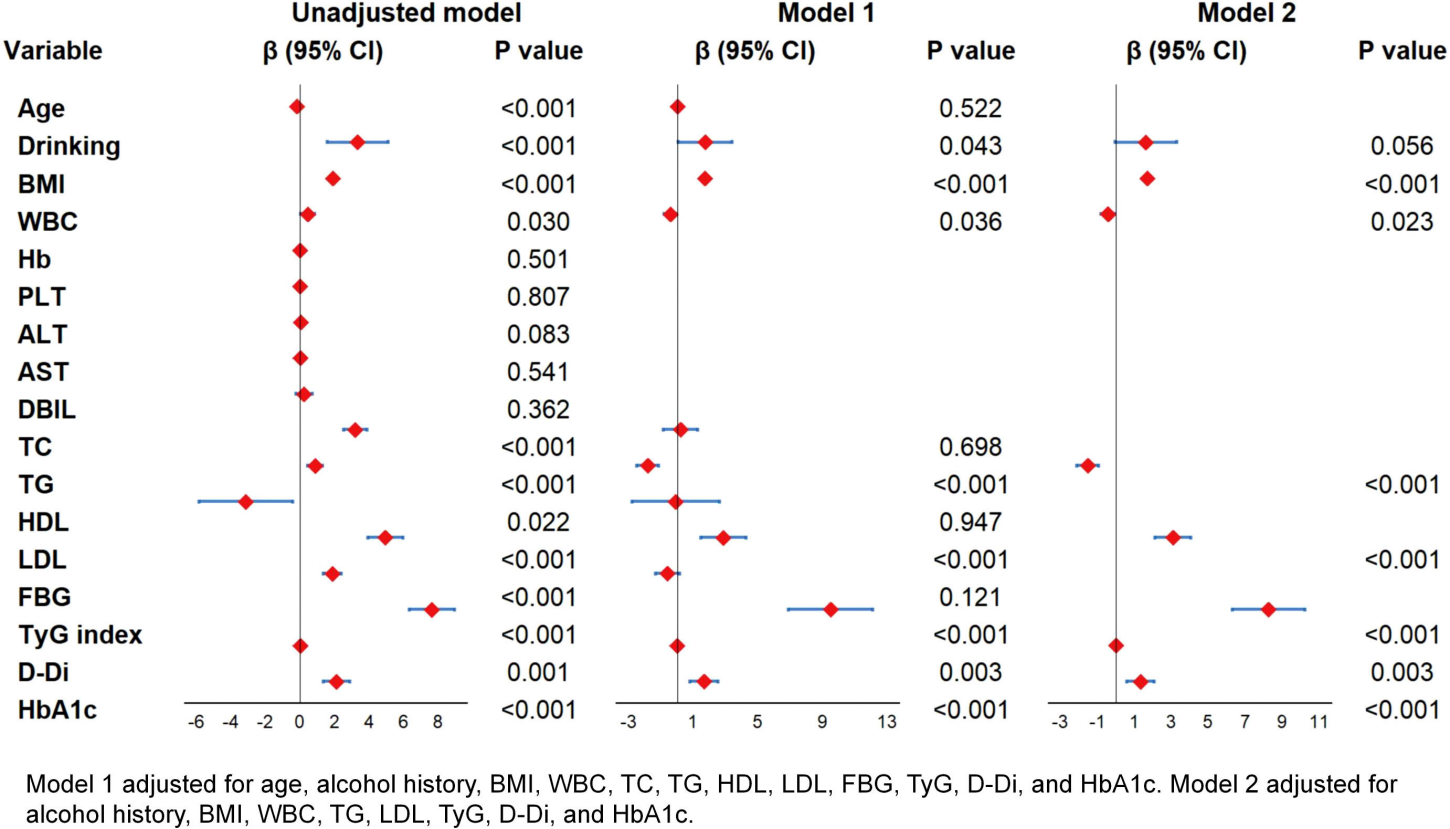
Univariate and multivariate linear regression of AHI.

Univariate analysis identified multiple factors associated with the apnea-hypopnea index, including age, alcohol consumption history, BMI, WBC, TC, TG, HDL, LDL, FBG, TyG index, D-Di, and HbA1c. Multivariate linear regression analysis revealed the TyG index as the strongest independent correlate (β=8.265, 95% CI:6.291-10.239; *P* < 0.001), demonstrating a linear dose-response relationship with AHI elevation - each unit increase in TyG index corresponded to an 8.265-unit rise in AHI. BMI emerged as the secondary significant determinant (β=1.702, 95% CI:1.523-1.882; *P* < 0.001), exhibiting 1.702-unit AHI elevation per BMI unit increment. While TyG and BMI demonstrated the strongest associations, additional contributors to AHI variation included WBC, TG, LDL, D-Di, and HbA1c, with all coefficients reaching statistical significance (*P* < 0.05) (**Figure 7**).

## Discussion

The TyG index, a recognized biomarker of insulin resistance, demonstrated independent association with OSA severity progression in this pioneering cohort of 562 patients with comorbid hypertension and OSA- a finding previously unreported in clinical literature. Multivariate ordinal logistic regression analysis revealed that each 1-unit elevation in TyG index increased the likelihood of advancing to higher OSA severity grades by 88.5%. Notably, the TyG index exhibited stronger associative strength compared to BMI, evidenced by both effect magnitude (OR ratio: 1.885 vs.1.110) and standardized β coefficients, suggesting insulin resistance may serve as the central pathogenic nexus bridging hypertension and OSA progression.

A growing body of clinical and epidemiological studies has confirmed the TyG index as a clinically relevant biomarker for OSA. A Meta-analysis that included 16,726 individuals showed a significant trend of elevated TyG index in patients with OSA compared to healthy controls, suggesting a potential correlation between TyG index and OSA (18). Notably, the triglyceride-glucose index demonstrates discriminative capacity in OSA diagnostic processes, achieving a peak receiver operating characteristic (ROC) curve area of 0.681, suggesting clinical viability for population-level risk stratification and case identification (18). Notably, prior investigations exhibited limited discriminative capacity across OSA severity strata, whereas a large-scale cohort analysis (n=4,703) demonstrated a monotonic TyG index escalation paralleling OSA progression gradients (19). Bikov et al (20) also confirmed a significant correlation between AHI and TyG index. The aforementioned studies have confirmed the association between TyG index and OSA. Numerous studies have suggested a close relationship between TyG index and hypertension. In a 9-year follow-up study, Longitudinal analysis spanning a decade revealed TyG index elevation independently predicted incident hypertension risk in Zheng’s cohort study (21). Lee et al (22) demonstrated a positive correlation between elevated TyG index and the risk of blood pressure progression in a Korean cohort comprising 15,721 normotensive adults. Zhu et al (23) reached similar conclusions in their investigation of 43,591 participants, further demonstrating that this association remained significant even after achieving optimal control of LDL-C or HDL-C levels. In this study, we also reached similar conclusions in hypertensive patients with OSA. In the univariate analysis, traditional metabolic indices like FBG and HbA1c showed correlations. Yet, after adjusting for confounding factors, only the TyG index remained significant, which confirms its unique superiority as a composite indicator in integrating dysglycemia and dyslipidemia.

The TyG index, a well-established marker of insulin resistance, highlights insulin resistance as a potential underlying mechanism for the comorbidity of hypertension and OSA. Prior studies have confirmed that OSA is independently associated with insulin resistance (24), and animal models of CIH further support this connection. CIH promotes oxidative stress and inflammation, elevating cytokines such as TNF-α, interleukin-6, and interleukin-18, which activate NF-κB and JNK pathways, impair insulin signaling, and contribute to endothelial dysfunction (25–27). Sympathetic overactivation in OSA also disrupts glucose regulation by increasing catecholamines and suppressing insulin secretion (28). Experimental evidence shows that CIH activates NF-κB and adhesion molecules, aggravating vascular injury (29, 30), while antioxidant interventions can normalize insulin resistance parameters (31). Together, insulin resistance, oxidative stress, sympathetic activation, and endothelial dysfunction constitute central mechanisms in OSA-related metabolic dysregulation.

Insulin resistance also plays a pivotal role in hypertension. Meta-analyses have shown it significantly increases hypertension risk (32, 33). Hyperglycemia and compensatory hyperinsulinemia expand circulatory volume, enhance sodium retention, and activate the renin–angiotensin system, while also sensitizing the carotid body to augment sympathetic outflow (32, 34, 35). Moreover, insulin normally promotes vasodilation through the PI3K/Akt–NO pathway (36) but also induces vasoconstriction via MAPK activation (37). In states of insulin resistance, this balance shifts toward vasoconstriction, vascular remodeling, and further blood pressure elevation through activation of the renin–angiotensin–aldosterone axis and endothelin synthesis (38). The TyG index, reflecting both glucose and lipid metabolism, thus serves as a clinically relevant biomarker for patients with coexisting OSA and hypertension.

Subgroup analysis revealed BMI-dependent variations in the associative strength of TyG index for OSA severity: a significant association was observed in non-obese individuals (BMI <30 kg/m²) with an OR of 2.804 (95% CI 1.547–5.083), whereas no statistical significance was detected in the obese group. This discrepancy suggests potential heterogeneity in OSA pathogenesis—insulin resistance may play a more prominent role in OSA development among non-obese populations, while other mechanisms likely dominate in obese individuals. These findings underscore the necessity of accounting for BMI when interpreting TyG index values to optimize OSA risk stratification across diverse patient subgroups. Interestingly, triglyceride levels showed a paradoxical protective association in non-obese OSA, which may be attributable to preserved adipose tissue functionality. Functional adipose tissue not only buffers lipid spillover but also acts as an endocrine organ; transplantation studies in rodents demonstrated that healthy adipose tissue reverses insulin resistance and improves glucose metabolism, whereas fat removal aggravates dysfunction (39, 40). Moreover, adipokines secreted by adipose tissue, particularly adiponectin, exert insulin-sensitizing and anti-inflammatory effects, with higher adiponectin levels being consistently associated with better metabolic status and lower systemic inflammation (41). Experimental models of chronic intermittent hypoxia further revealed tissue-specific adaptations in adiponectin receptor expression, suggesting compensatory lipid redistribution (42). Beyond metabolic regulation, adipose tissue also supports host defense through antimicrobial peptide secretion (43) and confers broader evolutionary advantages, including energy storage during starvation and reproductive success. Taken together, these findings highlight that in non-obese OSA patients, the quality and functionality of adipose tissue, together with preserved adiponectin sensitivity, may facilitate triglyceride uptake and storage, thereby explaining the inverse association with disease severity. Rather than reflecting the so-called “obesity paradox,” our findings underscore the possibility that an appropriate amount of functional adipose tissue may serve as a physiological protector of the human body.

In summary, the TyG index, a validated surrogate marker of insulin resistance, demonstrates significant associations with both OSA severity and hypertension development. This research examined the prognostic utility of the TyG index in hypertensive patients with coexisting OSA, aiding timely detection of at-risk populations and permitting specific preventive measures. Notwithstanding methodological robustness, certain constraints require consideration. Primarily, the observational study design limits causal interpretation of TyG-OSA severity associations, with possible bidirectional relationships (e.g., advanced OSA worsening metabolic dysregulation). Second, single-center recruitment may introduce selection bias. Third, the absence of detailed metabolic phenotyping data—including visceral adipose area and adiponectin levels—limits mechanistic exploration. Fourth, medication use such as antihypertensive, lipid-lowering, or antidiabetic agents was not systematically adjusted for in the analysis. As these drugs may influence glucose and lipid metabolism as well as the TyG index, their potential confounding effects cannot be excluded and should be considered in future studies. Future investigations should prioritize: (1) longitudinal studies elucidating the temporal relationship between TyG index and OSA incidence in prehypertensive populations; (2) mechanistic studies dissecting the interplay among insulin resistance, OSA, and hypertension; (3) clinical trials evaluating synergistic efficacy of TyG-guided therapies in ameliorating AHI.

## Conclusions

This study demonstrates the TyG index outperforms conventional obesity indicators in associating with OSA severity among patients with hypertension-OSA comorbidity, with enhanced predictive accuracy in non-obese subgroups. These findings clarify metabolic-inflammatory pathways in OSA pathogenesis and enable risk stratification using standardized biochemical parameters. Validation through comprehensive subgroup analyses supports clinical implementation of TyG-based monitoring for early intervention targeting cardiometabolic sequelae.

## Data Availability

The original contributions presented in the study are included in the article/supplementary material. Further inquiries can be directed to the corresponding author.
